# Alternative Isolation Protocol for Desulfo and Zwitterionic Cylindrospermopsin Alkaloids and Comparison of Their Toxicity in HepG2 Cells

**DOI:** 10.3390/molecules25133027

**Published:** 2020-07-02

**Authors:** Carlos González-Blanco, Felipe Augusto Dörr, Renata Albuquerque, Janice Onuki, Ernani Pinto

**Affiliations:** 1 Department of Clinical and Toxicological Analyses, School of Pharmaceutical Sciences, University of São Paulo, São Paulo 05508-900, SP, Brazil; cgonzalezb@poder-judicial.go.cr (C.G.-B.); fadorr@usp.br (F.A.D.); renatalb@usp.br (R.A.); 2 Laboratory of Development and Innovation, Butantan Institute, Av. Vital Brasil, 1500, São Paulo 05503-900, SP, Brazil; janice.onuki@butantan.gov.br; 3 Sección de Toxicología, Departamento de Ciencias Forenses, Organismo de Investigación Judicial, Heredia 40801, Costa Rica; 4 Centre for Nuclear Energy in Agriculture, University of São Paulo, Piracicaba 13416-000, SP, Brazil

**Keywords:** LC-MS^2^, LC-DAD, cylindrospermopsin, 7-deoxy-cylindrospermopsin, 7-deoxy-desulfo-cylindrospermopsin, cyanotoxins, HepG2 cells, biomass, culture broth

## Abstract

The term cylindrospermopsins (CYNs) refers to a structurally related class of cyanobacterial metabolites comprised of a tricyclic guanidine group and a hydroxymethyluracil moiety. Most reports in environmental aquatic samples refer to cylindrospermopsin (CYN), and reports on other CYN alkaloids are scarce, due, in part, to a lack of versatile isolation protocols. Thus, using commercially available solid phase extraction (SPE) cartridges, we optimized an isolation protocol for the complete recovery of CYN, 7-deoxy-cylindrospermopsin (7D-CYN) and 7-deoxy-desulfo-cylindrospermopsin (7D-desulfo-CYN) from the same aliquot. The isolation protocol was adaptable depending on the nature of the sample (solid biomass, culture broth or environmental water sample) and tolerates up to 4 L of dense culture broth or 400 mg of lyophilized biomass. To quantitate the CYN alkaloids, we validated an LC-DAD-MS^2^ method, which takes advantage of the UV absorption of the uracil group (λ 262 nm). Using electrospray ionization (ESI) in a positive ion mode, the high-resolution MS^1^ data confirms the presence of the protonated alkaloids, and the MS^2^ fragment assignment is reported as complementary proof of the molecular structure of the CYNs. We isolated three CYN alkaloids with different water solubility using the same lyophilized sample, with a purity that ranged from 95% to 99%. The biological activity of the purified CYNs, along with a synthetic degradation product of CYN (desulfo-cylindrospermopsin), was evaluated by assessing necrosis and apoptosis in vitro using flow cytometry. CYN’s lethal potency in HepG2 cells was greater than the other analogs, due to the presence of all four functional groups: guanidine, uracil, C-7 hydroxyl and the sulfate residue.

## 1. Introduction

The cyanotoxin cylindrospermopsin (CYN) is a worldwide health risk. *Cylindrospermopsis* (*Raphidiopsis) raciborskii* and other species capable of synthesizing this molecule have been reported in water bodies of tropical and temperate regions of Australia, North America, South America, New Zealand and Europe. However, cyanobacterial species capable of producing CYN and its structural variants are found in almost all of the world’s latitudes [1,2]. In Brazil, the problem of cyanobacteria and their toxins in water is widely known and is a matter of public health policies [3,4,5].

Exposure to CYN produces a fatty liver and hepatonecrosis in experimental animals, with extrahepatic lesions of variable location and severity. The main toxic effects appear to be irreversible protein synthesis inhibition, oxidative stress and deoxyribonucleic acid (DNA) damage. Nevertheless, comprehensive and specific mechanisms of toxicity are yet unclear [4,6].

**Figure 1:**
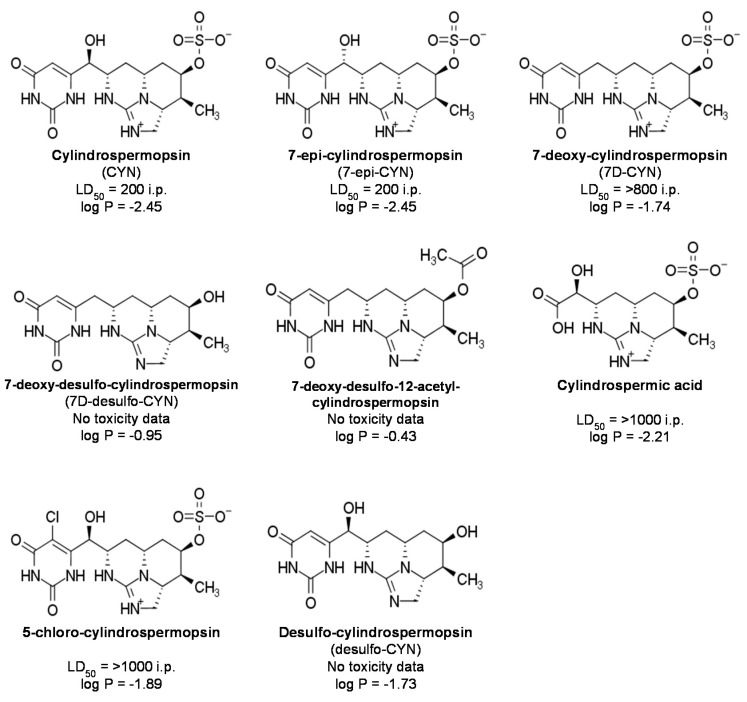
Structure, toxicity and theoretical log P of natural cyanotoxin cylindrospermopsin (CYN) alkaloids and degradation products. LD_50_ (µg/kg) refers to the median lethal dose in mice, administered by intraperitoneal (i.p.) injection. Theoretical log P corresponds to the arithmetic mean of the values calculated by online cheminformatics tools (see Table S1 for details) [7,8,9,10].

Cylindrospermopsins (CYNs) are structural analogs of the originally reported CYN molecule. Limited information is available on the distribution, occurrence and toxicity of other naturally occurring CYN-like secondary metabolites from cyanobacteria [4,6,11,12,13,14,15,16,17]. Figure 1 shows the CYNs reported to date, along with their reported toxicity and calculated octanol/water partition coefficient (log P). CYN, 7-epi-cylindrospermopsin (7-epi-CYN) and 7-deoxy-cylindrospermopsin (7D-CYN) are the main zwitterions reported in freshwater samples and laboratory strains [13,16,18]. These zwitterion variants consist of a negatively chargeable sulfate group, a positively chargeable tricyclic guanidine portion and a biologically active uracil group. In the case of CYN and 7-epi-CYN, the uracil moiety is bound to the heterocyclic rings by a hydroxylated asymmetric carbon. This hydroxylation is absent in 7D-CYN, as well as in 7D-desulfo-CYN and 7-deoxy-desulfo-12-acetyl-cylindrospermopsin. Additionally, the absence of the sulfate anion on these last two desulfo variants makes them less hydrophilic compared to the zwitterion variants. Figure 1 also shows the structure of cylindrospermic acid and 5-chloro-cylindrospermopsin, two innocuous oxidation products reported in the literature. Desulfo-cylindrospermopsin (desulfo-CYN) is a degradation product that can be formed by acid hydrolysis.

Both 7D-CYN and 7D-desulfo-CYN are biosynthetic precursors of the originally reported CYN molecule. The functional guanidino moiety is the starting unit in the biosynthesis of the carbon backbone of CYN. An uncommon biosynthetic pathway of pyrimidine is responsible for the de novo formation of the uracil ring. Finally, sulfation and hydroxylation tailoring reactions add the last modifications that culminate in the fully bioactive cylindrospermopsin molecule [19,20].

Cylindrospermopsin is a widely studied molecule for which numerous extraction protocols and toxicity assays have been described. 7D-CYN is a zwitterion analog, structurally similar enough to be simultaneously isolated along with CYN in most frequently used extraction protocols [11,13,21,22]. However, other CYN-like metabolites are less reported. In 2014, Wimmer et al. reported the natural occurrence of two alkaloids with different hydrophobicity than the previously reported CYNs: 7D-desulfo-CYN and 7-deoxy-desulfo-12-acetylcylindrospermopsin [15]. At present, there are no certified standards for 7D-desulfo-CYN and 7-deoxy-desulfo-12-acetylcylindrospermopsin. 7D-CYN-certified standards are available by several vendors. Nevertheless, 7D-CYN standards are currently sold in low concentrations that are inappropriate for the validation of analytical methods or biological testing purposes.

The chemical properties of the less-hydrophilic CYNs indicate that they may accumulate in other matrixes other than freshwater—for example, inside cyanobacterial cells, animal tissue, sediments or water filter resins. The distribution of desulfo CYNs in ecological systems is unknown.

7D-desulfo-CYN serves as an important analyte in order to study the importance of the C-7 hydroxylation in the lethal potency of CYN alkaloids that lack the sulfate group. Another desulfo analog, desulfo-CYN, can be synthesized by the acid hydrolysis of CYN at a constant temperature. Reports on the acute toxicity of desulfated CYNs are scarce, and the chronic effects of these desulfo variants are far from being elucidated. Here, we report a novel SPE workflow to isolate cylindrospermopsins (CYN, 7D-CYN and 7D-desulfo-CYN) simultaneously from the same aliquot of a natural producing strain in order to assess their lethal potencies on HepG2 cells.

## 2. Results

### 2.1. Tests on Sample Size and Cartridge Handling

According to previous analysis from our group, the *C. raciborskii* 11K strain produces high quantities of CYN and 7D-CYN, a great part of which is secreted to the culture medium (data not shown). There was also evidence of a third CYN natural alkaloid, which was later confirmed as 7D-desulfo-CYN. In order to selectively isolate these metabolites, our initial approach was to separate them from a simple liquid matrix (filtered culture medium) using SPE cartridges. Subsequently, we tested the performance of SPE sorbents on a more complex solid matrix (lyophilized culture biomass).

#### 2.1.1. Liquid Samples

CYN and 7D-CYN are heavily secreted and are available in high quantities in the culture medium of *C. racibosrkii* 11k. As stated earlier, 7D-desulfo-CYN is less hydrophilic, and it is scarce in the aqueous culture medium.

Small-scale experiments using liquid medium were performed initially in the liquid sample to assess the adsorption of the analytes (mostly CYN and 7D-CYN) on two SPE columns: graphitized nonporous carbon (GNPC) and C18 (Figures S1–S5). The adsorbed analytes were eluted using four different methanol (MeOH) concentrations (Figures S2–S5). CYN and 7D-CYN showed high affinity for the GNPC stationary phase, and it became necessary to elute the carbon cartridges in backflush mode. Backflush elution refers to the reverse use of the SPE cartridge, where the liquid flow enters the cartridge through the bottom frit and exits by the top frit.

The GNPC cartridge is not particularly designed for backflush mode, although it can be improvised. Nevertheless, eluting with various MeOH concentrations with the GNPC cartridge in backflush mode was challenging. Thus, we tested a modified version of the elution technique reported by Norris et al. (2001) [17]. In this manner, we used MeOH:CH_2_Cl_2_ 4:1 with 5% formic acid (FA) to elute the analytes without needing to backflush the cartridge. Table S2 shows the percent recovery of the three CYN variants when extracted from GNPC cartridges using two kinds of elution techniques. The elution performance for CYN using MeOH:CH_2_Cl_2_ 4:1 (*v*/*v*) with 5% formic acid yielded a better percent recovery than the backflush-mode technique. Elution using MeOH:CH_2_Cl_2_ 4:1 with 5% FA needed no adaptation, as is required when eluting in backflush mode. Since the MeOH:CH_2_Cl_2_ 4:1 with 5% FA elution was easier and more efficient for cylindrospermopsin, it was chosen as the elution method for the subsequent isolation experiments.

#### 2.1.2. Solid Samples

Compared to the liquid medium, the remaining biomass from the broth extraction experiments contained the three CYN variants in higher concentrations. The recovered biomass comprises mostly on intact cyanobacteria separated by centrifugation, and it constitutes a complex matrix compared to the broth. Compared to the culture medium, the less hydrophilic alkaloid 7D-desulfo-CYN is stocked in higher concentrations inside the cells. In order to recover a cleaner extract of 7D-desulfo-CYN, we decided to include an extra SPE step before the GNPC extraction.

Therefore, we tested the affinity of 7D-desulfo-CYN to C18 and HLB cartridges using the remaining biomass from the liquid samples experiments (Materials and Methods 4.3). HLB and C18 cartridges retained 7D-desulfo-CYN with similar performances at the samples’ natural pH (7.5 ± 0.5). Elution of the cartridges with 10 mL of 15% MeOH selectively rinsed CYN and 7D-CYN from the column’s sorbent. The following fraction eluted with 100% MeOH contained the 7D-desulfo-CYN analog. Fewer contaminants were detected in this last elution step while using C18 columns.

In this manner, the aqueous extract from the lyophilized biomass requires an extra step involving a C18 cartridge to retain 7D-desulfo-CYN. Next, an elution with 15% MeOH is needed to rinse CYN zwitterions and other highly hydrophilic metabolites from the C18 column into another cartridge. A GNPC cartridge can be adapted in series to the C18 column to adsorb CYN and 7D-CYN, as reported by Wimmer et al. (2014) [15].

**Figure 2:**
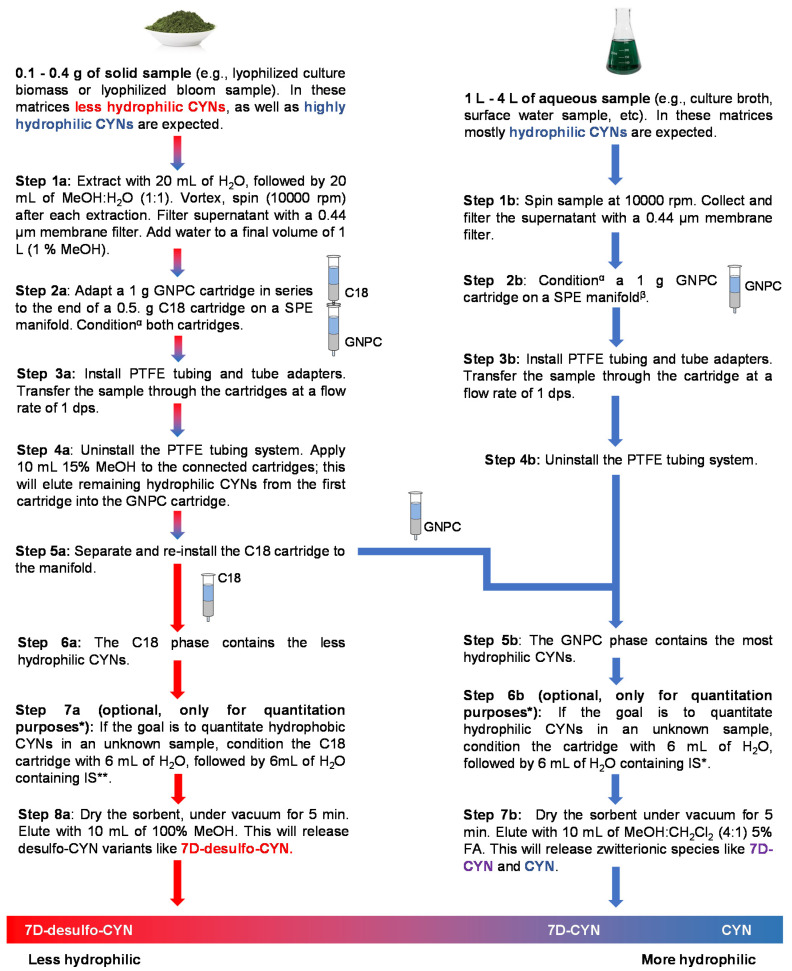
Proposed workflow for the simultaneous purification of zwitterionic (more hydrophilic) and desulfo (less hydrophilic) cylindrospermopsin analogs from cyanobacteria biomass, culture broth or surface water samples. MeOH: methanol, SPE: solid-phase extraction, GNPC: graphitized nonporous carbon, PTFE: polytetrafluoroethylene, IS: internal standard, CH_2_Cl_2_: dichloromethane, FA: formic acid and dps: drop per second. ^α^Condition both cartridges with MeOH, followed by deionized H_2_O. Do not let the sorbents dry up in this step. ^β^Less-hydrophilic desulfo CYNs on aqueous samples are negligible; therefore, C18 cartridges are not necessary. * The IS must be added at this point if the extraction’s objective is the simultaneous quantitation of zwitterionic and desulfo CYNs. Spiking solid samples with IS is not recommended for this protocol, because acyclovir (IS) adsorbs to both sorbents (GNPC and C18). The resulting uneven distribution of the IS in both sorbents impedes the simultaneous quantitation of the CYN variants. ** Acyclovir was used as the IS.

Preliminary tests using acyclovir as internal standard (IS) showed a good affinity for the GNPC cartridge and C18 column (data not shown). Usually, the IS is applied directly to the intact sample. Nevertheless, in the case of solid samples, acyclovir can be partially eluted during Step 4a of the extraction protocol (Figure 2). We proposed adding the IS directly to the SPE columns (Figure 2, Steps 7a and 6b) for the protocol to be compatible with quantitative analysis. The IS was added in this manner during the validation experiments, as described in Materials and Methods (4.5–4.6).

Finally, a summarized version of the whole workflow is displayed in Figure 2. The capacity of the tested SPE columns allows handling up to 0.4 g of lyophilized cells or 4 L of filtered broth. The workflow was designed for the purification or the absolute quantitation of CYN variants simultaneously in the same sample.

### 2.2. Validation of the LC-DAD Method for CYNs Quantitation

CYN alkaloids produced by the *C. raciborskii* 11K strain display an uracil moiety, which absorbs UV radiation at a λ_max_ (maximum wavelength) of 262 nm. In order to quantify CYNs available in the samples, we took advantage of this shared functional group. At the time the experiments were designed, only CYN-certified standards were commercially available in enough quantities to perform validation protocols. Liquid sample extracts used for validation experiments were prepared as described in Figure 2, Steps 1b–7b. The validation guidelines and the liquid chromatography coupled to a diode-array detector (LC-DAD) method parameters are described in Materials and Methods, items 4.6 and 4.7, respectively.

Table S3 shows the experimental results and criteria related to the calibration, limit of detection (LOD) and lower limit of quantitation (LOQ). Within-day precision and bias calculations are displayed in Table S4. All analytical performance parameters were in accordance with the regulatory guidance requirements (Materials and Methods 4.6).

Selectivity was assessed by testing for matrix effects and by calculating the peak resolution (Rs) from the UV-extracted chromatogram at λ 262 nm. The matrix effect was evaluated by investigating the presence of interference signals (on the analytes’ retention times) using extracts from blank samples. Regarding the matrix effect, all tested samples were considered acceptable according to the criteria indicated in Materials and Methods 4.6. Likewise, chromatographic selectivity was assessed by calculating Rs for CYN and four other analytes in a nonextracted mix. Figure S8 shows a suitable peak resolution (R_S_ ≥ 2) for CYN, 7D-CYN, 7D-desulfo-CYN, desulfo cylindrospermopsin (desulfo-CYN) and the IS (acyclovir). Desulfo-CYN is an in-house synthesized degradation product of CYN, developed as detailed in Materials and Methods 4.6.

The uracil moiety is a requisite for the quantitation of CYN-like metabolites using the validated LC-DAD method. The hydroxymethyluracil functional group is essential for CYN’s toxicity and is present in all of the natural CYNs described to date, unlike the sulfate group [12,13,15,16,23,24,25]. The quantitation of 7D-CYN, 7D-desulfo-CYN and desulfo-CYN was carried out by interpolating their normalized peak area into the calibration curve of the cylindrospermopsin-certified standard.

### 2.3. Identity and Purity of Isolated CYN Analogs

All CYN structural variants extracted by SPE underwent an extra LC-DAD purification step with a C18 semipreparative column using a chromatographic method described in the Materials and Methods (item 4.4). This extra purification step was needed in order to separate CYN and 7D-CYN from the same SPE fraction and eliminate the impurities. On the other hand, the 7D-desulfo-CYN isolated with C18 cartridges had few impurities left. Nevertheless, it was also subjected to the same LC-DAD isolation step.

#### 2.3.1. Purity Assessment Using LC-DAD-MS^2^

**Figure 3:**
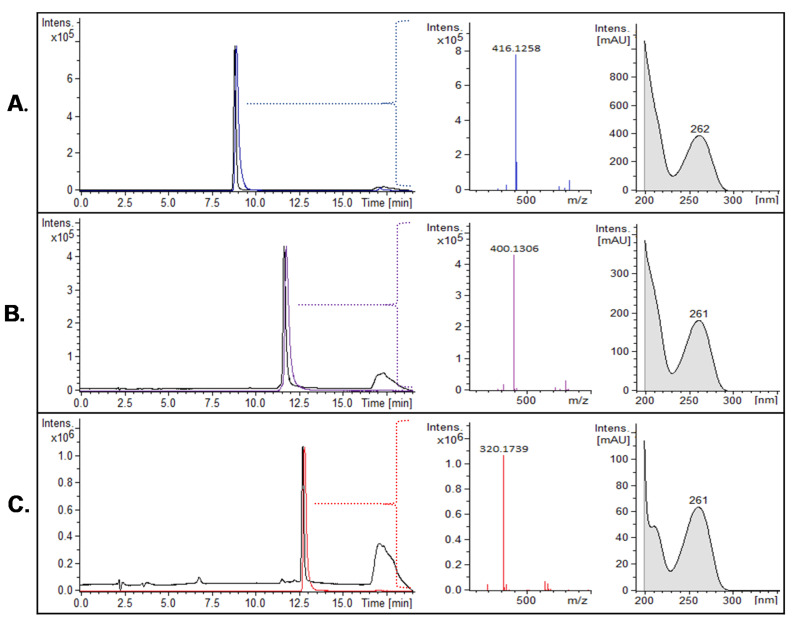
Purity assessment of three isolated CYNs: (**A**) CYN, (**B**) 7D-CYN and (**C**) 7D-desulfo-CYN. Every subfigure shows, in sequential order: overlapped EIC * (colored chromatogram) and EC ** at λ 262 nm (black chromatogram), the protonated precursor’s *m*/*z* (colored mass spectrum) and the UV absorption spectrum. * EIC: extracted ion chromatogram (MS) of the protonated precursor ion. ** EC: extracted chromatogram (UV). mAU: milli-absorbance unit.

The purity of the isolated CYN analogs was determined from the UV-extracted chromatogram at λ 262 nm. The purity of the isolated CYNs ranged from 97% to 99%. Figure 3 shows an example of three lots of purified alkaloids, analyzed by liquid chromatography coupled to a diode-array detector and connected to a tandem mass spectrometer (LC-DAD-MS^2^). The extracted ion chromatogram (EIC) of the protonated precursor ion (acquired by the quadrupole time-of-flight—QTOF—analyzer) and the UV spectrum (acquired by the diode-array detector) confirm the identity of each purified CYN analog (Figure 3).

#### 2.3.2. Molecular Structure Confirmation Using LC-DAD-MS^2^

All three naturally occurring CYN alkaloids (Table 1) were identified with mass accuracy <1.6 ppm, considering the mass error of the protonated precursor ion (MS^1^). These measures were performed on different days (n = 3).

**Figure 4:**
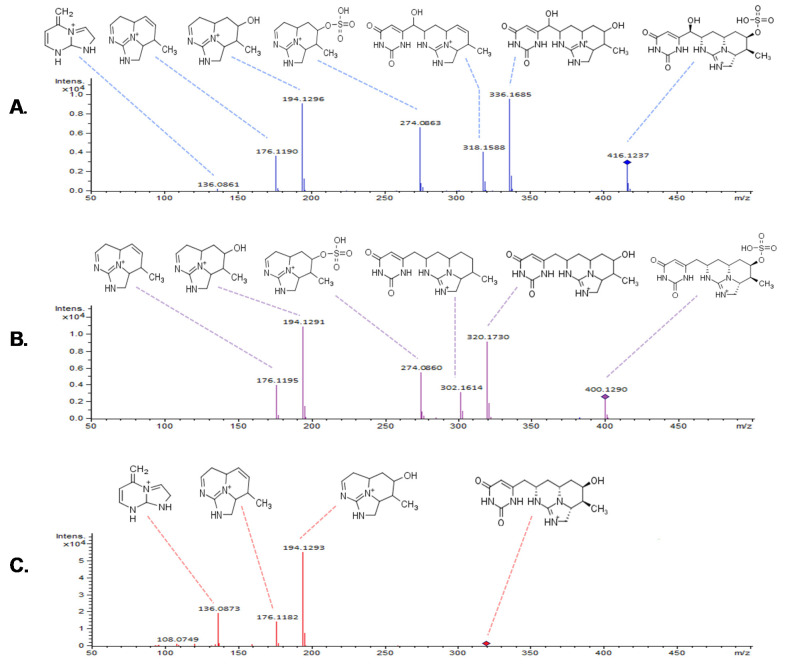
Proposed structure assignment of CYNs fragmentation patterns on a QTOF mass analyzer: (**A**) CYN at 20 eV, (**B**) 7D-CYN at 20 eV and (**C**) 7D-desulfo-CYN at 35 eV. Structure assignment was based on previous calculations by Dörr et al. (2010) [26].

The fragmentation pattern of CYNs in the gas phase was studied using the three purified metabolites. The QTOF mass analyzer was set in multiple reaction monitoring (MRM) mode to perform collision-induced dissociation (CID) experiments at the following collision energies: 20 eV, 35 eV and 40 eV. The collision energies included in Figure 4 generated a reproducible and rich mass spectra.

A partial assignment of adducts and fragments of CYN and 7D-CYN was described by Norris et al. (1999) [13]. Later, ion trap fragmentation studies of CYN metallic adducts confirmed the CID (collision-induced dissociation) reactions that precursors underwent in the gas phase of the mass analyzer [26]. The structure of the most intense MS^2^ ions of 7D-CYN and 7D-desulfo-CYN (Figure 4) was successfully assigned based solely on the gas-phase fragmentation reactions proposed by Dörr et al. (2008). The resulting spectra were useful in order to compare the fragmentation patterns of 7D-CYN and 7D-desulfo-CYN (Figure 4).

#### 2.3.3. Toxicity Assessment Using Flow Cytometry

Previously reported cell proliferation testing using MTT (3-(4,5-dimethylthiazol-2-yl)-2,5-diphenyltetrazolium bromide) gave us a starting point from which to choose appropriate concentrations of CYNs for the cell stimulation. The MTT assay evaluates the mitochondrial dehydrogenase activity of cells by measuring the formation of formazan crystals. In one study, the MTT assay yielded an IC_50_ (half-maximal inhibitory concentration) of 1.5 ± 0.9 µM after a 24-h stimulation with CYN in HepG2 cells [27].

Likewise, two studies found no significant differences between the vehicle control and CYN-treated HepG2 cells at 0.5 μg/mL [28] and 2 μg/mL [29] after 24 h.

In this work, we initially required the calculation of the threshold concentrations of the CYN analytical standard that significantly decreased HepG2 cells’ viability in the relevant time intervals. Next, we wanted to use the previously defined threshold concentrations to treat HepG2 cells with the purified CYNs (to calculate their toxicity). The effects of the purified CYN variants on HepG2′s viability measured by flow cytometry could later be compared to previously published data to evaluate the isolated CYN variants’ potency.

**Figure 5:**
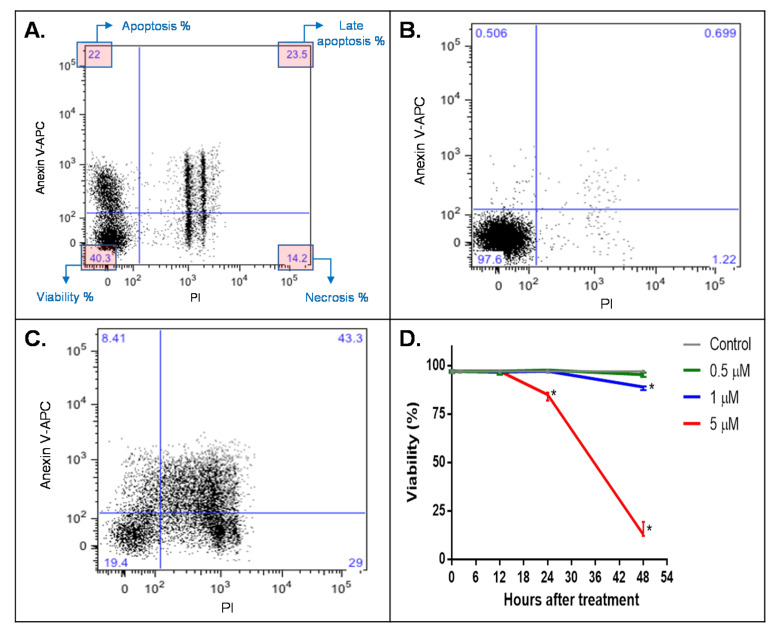
Biological activity of the CYN-certified standard (Abraxis^®^) on HepG2 cells, analyzed by flow cytometry using propidium iodide (PI) as the necrosis marker and annexin V-APC as the apoptosis marker: (**A**) Example of viability percentage (%) of a 24-h apoptosis and necrosis positive control. (**B**) Example of a 24-h negative control. (**C**) Example of a 48-h treatment with 5-µM CYN. (**D**) Viability after treatment using 0.5, 1 and 5 µM of CYN for 12, 24 and 48 h (n = 4). APC: allophycocyanin; statistical comparisons between groups (control vs. treatment) were performed using Mann-Whitney U tests; *p* < 0.05. * Statistically significant group compared to the control group.

For this purpose, HepG2 cells were stimulated with different concentrations of the CYN analytical standard (Abraxis^®^) for up to 48 h (Figure 5D). No significant viability decrease was detected by the flow cytometry assay on HepG2 cells using 1 µM of CYN for a 24-h time interval. In a 48-h treatment, a stimulation with 0.5 µM of CYN did not induce apoptotic nor necrotic effects, while higher concentrations (1 µM and 5 µM) caused a significant viability decrease on the cell cultures.

**Figure 6:**
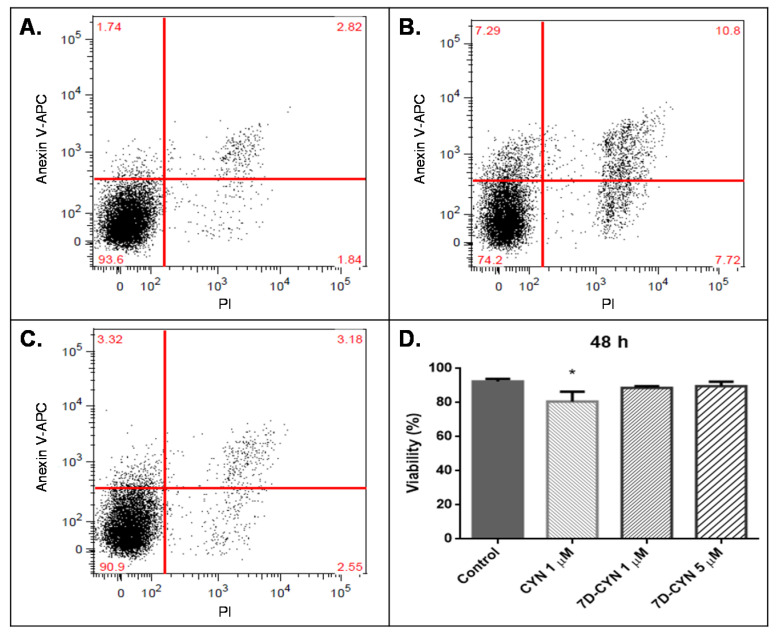
Biological activity of CYN and 7D-CYN purified from *C. raciborskii 11K* on HepG2 cells analyzed by flow cytometry using propidium iodide (PI) as the necrosis marker and annexin V-APC as the apoptosis marker: (**A**) Example of a 48-h negative control. (**B**) Example of a 48-h treatment with 1 µM CYN. (**C**) Example of a 48-h treatment with 5 µM 7D-CYN. (**D**) Viability after treatment using 1 µM CYN and 1 and 5 µM 7D-CYN (n = 4). APC: allophycocyanin; statistical comparisons between groups (control vs. treatment) were performed using Mann-Whitney U tests; *p* < 0.05. * Statistically significant group compared to the control group.

**Figure 7:**
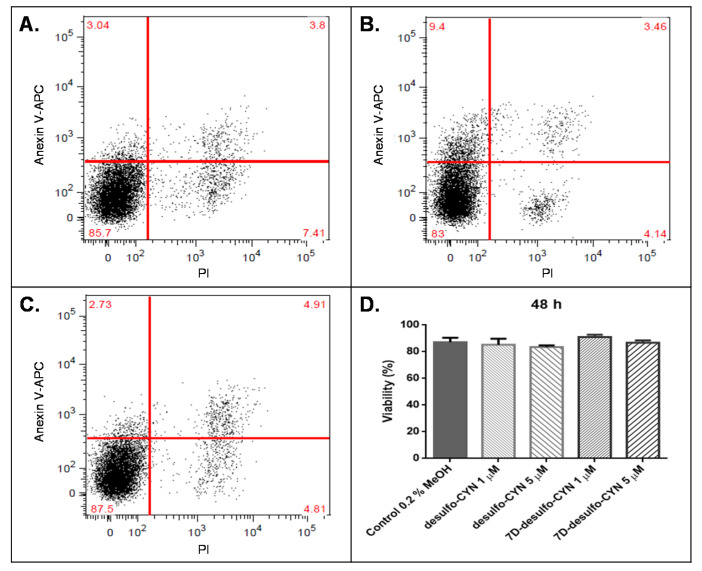
Biological activity of desulfo-CYN and 7D-desulfo-CYN purified from *C. raciborskii 11K* on HepG2 cells analyzed by flow cytometry using propidium iodide (PI) as the necrosis marker and annexin V-APC as the apoptosis marker. (**A**) Example of a 48-h negative control 0.2% MeOH. (**B**) Example of a 48-h treatment with 5 µM desulfo-CYN. (**C**) Example of a 48-h treatment with 5 µM 7D-desulfo-CYN. (**D**) Viability after a 48-h treatment using 1 and 5 µM of desulfo-CYN and 7D-desulfo-CYN (n = 4). APC: allophycocyanin signal; statistical comparisons between groups (control vs. treatment) were performed using Mann-Whitney U tests; *p* < 0.05.

In the same manner, we treated HepG2 cells with the purified CYN alkaloids (Figure 6 and Figure 7) to compare their potency against the commercial CYN standard. We also tested the biological activity of the degradation product desulfo-CYN (Figure 7).

Figure 6D indicates that, after a 48-h treatment, the purified CYN standard (lot 2016.07.03) showed a lethality on HepG2 cells compared to the lethality of the certified CYN standard (Abraxis^®^).

On the other hand, cells stimulated with 7D-CYN, 7D-desulfo-CYN and desulfo-CYN showed no significant difference in viability compared to their respective controls at the tested conditions (Figure 6 and Figure 7).

## 3. Discussion

We present in this work new comparative toxicological studies in vitro of zwitterionic and desulfated CYN alkaloids. Comparative studies between naturally occurring CYN alkaloids are not common, mainly due to a lack of popular and accepted extraction protocols for these metabolites.

The workflow described in Figure 2 aims to improve the monitoring of CYNs by concurrently isolating CYN variants with different water solubilities, depending on the nature of the sample. As evidenced in Figures S1–S7, considerable effort was invested in testing different SPE sorbents’ abilities to retain the CYN analogs after applying various elution techniques. Our main concern was to implement efficient and selective elutions on the ideal sorbent, while ensuring the maximum recovery of each analyte.

Norris et al. (2001) documented the benefits of using graphitized carbon cartridges (instead of C18) to retain CYN zwitterions [17]. Nevertheless, C18 SPE columns proved optimal in order to selectively adsorb less-hydrophilic CYNs [15]. The coupling of these two SPE techniques while using selective eluents can improve the recovery of each unique CYN-like molecule.

Unknown solid samples composed mostly of the biomass may contain highly hydrophilic and, also, less-hydrophilic CYNs. As shown in Figure 2 (Step 2a), after the application of the sample to the coupled cartridges, less-hydrophilic variants remain in the C18 phase, while zwitterions adsorb mainly on GNPC. Elution with 15% MeOH efficiently releases the remaining zwitterions from the C18 phase, and these, in turn, adsorb to the GNPC cartridge. Desulfo-CYNs in an unknown sample will remain in the C18 phase and can be subsequently eluted with pure MeOH. On the other hand, hydrophilic zwitterions bond strongly to the GNPC phase, and pure acidified MeOH is not sufficient to completely release these metabolites from the solid phase. However, the complete recovery of zwitterions from the carbon phase was reliably achieved with 10 mL of MeOH:CH_2_Cl_2_ 4:1 with 5% FA; there was no need for successive elutions, even when extracting 0.4 g of the biomass from our toxin-producing strain.

The validation of the protocol only focused on CYN, due to the lack (at the time) of commercially available certified standards of 7D-CYN at concentrations useful to prepare spiked samples. As stated earlier, no certified standards of 7D-desulfo-CYN (or other desulfo alkaloids) are currently available. Consequently, we decided to only include in the validation plan steps 1b to 7b of the workflow (Figure 2). As indicated in the Results (Section 2.2) and Supplementary Materials (Figures S8 and S9 and Tables S3 and S4), all the analytical performance parameters evaluated in the validation were under regulatory guidance requirements.

After confirming the quantity, purity and structure of the metabolites, we carried out toxicity assays on HepG2 cells. Fluorescence-activated cell sorting (FACS) flow cytometry was chosen as the ideal technique to test the purified CYNs’ toxicity. Some benefits of using FACS are that it monitors a representative sample of our cells (10,000 events), it is selective enough to identify the type of cell death (necrosis or apoptosis) and it is a highly sensitive technique because of the use of fluorescent markers.

Toxicity data resulting from our experiments matched the CYNs’ cell death results reported by Štraser et al. (2013) using annexin V and PI staining for FACS analysis. Their research found no significant difference in cell death between the vehicle control and treated HepG2 cells at 0.5 μg/mL for 12 and 24 h [30]. Using a similar methodology (Figure 5D), we did not find a significant difference in cell death between the control (100% viability) and cells treated with 1 µM (0.4 μg/mL) of the certified CYN standard during 12 and 24 h. Additionally consistent with Štraser et al.’s work, significant cell death was detected after a 48-h stimulation with 1 µM of the CYN-certified standard (Figure 5D) and 1 µM of purified CYN (Figure 6D). Intriguingly, a two-h treatment with 381 µM of CYN showed no significant viability decrease (data not shown). This was the highest concentration we could prepare with the available CYN-certified standard. Even though it is not a practical working concentration, the results with 381 µM of CYN served as evidence of the cyanotoxin’s delayed toxicity. In other words, the toxin’s apoptotic and/or necrotic effects are not immediate to HepG2 cells even at high concentrations.

In our current work, 7D-CYN caused no significant loss in viability at 1 µM (0.4 μg/mL) and 5 µM (2.0 μg/mL) after a 48-h treatment (Figure 6D). These results are consistent with reports by Neumann et al. (2007). Their group implemented a cell proliferation assay using the 3-(4,5-dimethylthiazol-2-yl)-5-(-3-carboxymethoxyphenyl)-2-(4-sulfophenyl)-2H-tetrazolium inner salt (MTS) and the electron-coupling reagent phenazine ethosulfate (PES). They stimulated with 7D-CYN several cell lines—of which, HepG2 was the least affected—yielding an IC_50_ of 3.2 μg/mL at 24 h and 3.1 μg/mL at 48 h [12]. According to Looper et al. (2005), 7D-CYN is a potent inhibitor of protein synthesis in vitro. Nevertheless, a complete protein synthesis inhibition required 10 µM in rat hepatocytes, a concentration higher than the ones tested in our current work [24].

Finally, we found that 7D-desulfo-CYN caused no significant cell death compared to the vehicle control at 1 µM (0.4 μg/mL) and 5 µM (2.0 μg/mL) after a 48-h treatment (Figure 7D). In the same manner, no significant viability loss was detected using 1 µM and 5 µM of the in-house degradation product desulfo-CYN. The vehicle control cells were grown in culture medium containing 0.2% MeOH to account for the methanol content of the purified desulfo analogs’ fractions.

Several authors have stated the essential role of the guanidine, uracil and hydroxyl groups in the toxic mechanism of CYN [23,31,32,33]. On the other hand, it has been proposed that the sulfate group of CYN has no role in its toxicity or its transport through the rat hepatocyte’s membrane. According to Runnegar et al., the sulfate group at C-12 of CYN is not required to induce a protein synthesis inhibition, nor is it required for cellular uptake in primary rat hepatocytes (PRH) [31]. In the current work, no significant apoptosis nor necrosis was detected in HepG2 cells after stimulation with up to 5 µM of two different desulfo variants. This incongruence can be explained by taking into account the cell type (PRH) and, also, the kind of biological activity assays (protein synthesis and cellular glutathione quantitation) used by Runnegar et al. (2002). Direct and indirect protein synthesis in vitro measurements are sensitive and specific of CYN alkaloids’ biological activity; these tests may not correlate well with cell viability assays (detection of the onset of necrosis and apoptosis). Likewise, PRH are known to be more sensitive to the toxic mechanisms of CYN. PRH display a wide variety of metabolic enzymes, membrane receptors and active transport proteins that resemble the liver parenchyma [6,34,35]. The HepG2 hepatoma cell line lacks this diversity, making it less sensitive to the toxicity of CYN alkaloids [12,35]. Taking this into account, it is likely that higher doses of desulfo-CYN and 7D-desulfo-CYN are necessary to detect their lethal potency in HepG2 cells.

Interestingly, 7-deoxy-desulfo-CYNs tend to be less-hydrophilic than the zwitterionic alkaloids (according to the n-octanol and water partition coefficients in Figure 1). Lipophilicity, commonly represented by the log P value, is the definitive factor in a molecule’s absorption, diffusion across cellular and tissue membranes, metabolism and excretion. Thus, a correlation between the lipophilicity and biological activity of small molecules is usually made [36,37]. In this way, the deoxy-desulfo analogs are favored with a better chance of passive membrane transport. Nevertheless, the enhanced capacity of desulfated analogs to penetrate cell membranes in comparison to zwitterionic species was not reflected in their lethality towards HepG2 cells. Only the original cylindrospermopsin alkaloid was able to significantly decrease viability in the hepatoma cell line at the tested conditions.

It has been demonstrated that 7D-desulfo-CYN and 7D-CYN correspond to the last two biosynthetic steps of CYN [19]. In the last biosynthetic step, the cyrI gene of the cyr cluster encodes a 2-oxoglutarate-dependent hydroxylase that transforms 7-deoxy-cylindrospermopsin into the diastereomers CYN and 7-epi-CYN [20]. The pharmacological significance of the C-7 hydroxyl group lies in being the last modification of a complex biosynthetic pathway in order to enable its biological activity. Previously, synthetic CYN analogs were used by other authors to test the pharmacological importance of the C-7 hydroxyl group [32,33]. Studies by Cartmell et al. (2017) compared the bioactivity of several synthetic analogs, revealing that the hydroxyl group at C-7 was indispensable in causing significant toxicity in human cells. The authors proposed that the C-7 hydroxyl group may be crucial for the active cellular transportation of CYN, analogous to the uptake of cholesterol. Evans et al. (2019) tested the biological activity of new synthetic CYN analogs with variations in their functional groups but all possessing the uracil ring. These experiments showed that the C-7 hydroxyl group, as well as the guanidine moiety, have a role in cylindrospermopsin’s toxicity and that both are essential for the molecule’s biological activity [33].

The toxicity assays presented in this work indicate that, in our biological model, a combination of the guanidino, uracil, sulfate and C-7 hydroxyl functional groups is necessary for a CYN analog to efficiently induce cell death. Future investigations using desulfo CYN variants may include toxicity tests using 3D hepatocyte cultures, toxicity tests on PRH cultures and in vivo toxicokinetics studies. The isolation workflow proposed by our group may serve useful for the forthcoming research of uncommon CYN alkaloids.

## 4. Materials and Methods

### 4.1. Cylindrospermopsis raciborskii Strain Maintenance

The *Cylindrospermopsis raciborskii* strain 11K is an Australian strain provided by Professor Marli Fatima Fiori at the Centro de Energía Nuclear na Agricultura (CENA), Universidade de São Paulo (Campus Luiz de Queiroz, Piracicaba). This strain produces CYN, 7D-CYN and 7D-deoxy-CYN. A Brazilian non-CYN-producing strain (*C. raciborskii* ITEP 18) was grown and used as a matrix for CYN spiked-in extractions during the validation of the LC-DAD method. These strains were maintained in our laboratory using the ASM-1 culture medium proposed by Gorham et al. (1964), with some modifications [38,39].

Both cyanobacterial strains were cultured inside an incubator at the desired final volume, with a working temperature of 22 °C, a 12-h light/dark interval and a light intensity of 30 to 50 μmol. photons.m^−2^.s^−1^;calibrated by a LI-COR^®^ 250A (LI-COR, Lincoln, NE, USA) light meter.

### 4.2. Tests on Sample Size and Cartridge Handling for Liquid Samples

Extraction experiments to assess the adsorption of the analytes in two different SPE sorbents required liquid samples obtained from the *Cylindrospermopsis raciborskii* strain 11K cultures with a density of 1 × 10^8^ cells/mL.

Each lot of approximately 20 L of dense culture broth was centrifuged (10,000 rpm, 10 min, 4 °C), and the supernatant (liquid medium) was collected. The remaining precipitate (filamentous cells or cellular debris) was frozen at −20 °C before lyophilization. Next, we filtered the culture medium through a 0.44-µm membrane to avoid clogging of the SPE cartridges.

Small-scale experiments using the liquid medium were performed initially to assess the adsorption of the analytes (mostly CYN and 7D-CYN) on two SPE columns: C18 Sep-Pak 1g (Waters^®^, Milford, MA, USA) and graphitized nonporous carbon (GNPC) Envi-carb 1g (Supelco^®^, Bellefonte, PA, USA). On the first experiment, SPE columns were pretreated with 10 mL of MeOH, followed by 10 mL of water. After the SPE column conditioning, we applied 300 mL of liquid medium to each cartridge through a PTFE (polytetrafluoroethylene) tubing system connected by a SPE tube adapter. The adsorbed analytes were eluted using 5 mL of each of the following aqueous solutions: 10%, 50%, 75% and 100% MeOH (Figures S1–S5). The GNPC cartridges were eluted in backflush mode (or reverse mode) due to the high affinity of the analytes to the stationary phase. The recovered volumes were dried and concentrated in 200-µL 50% MeOH before LC-DAD analysis.

Another small-scale experiment using the liquid medium was aimed to evaluate the percent recovery of CYNs using two elution techniques for the GNPC cartridges. GNPC columns were pretreated initially with 10 mL of MeOH. After this step, we applied 10 mL of water to each SPE column. Once the SPE columns were conditioned, we applied 300 mL of the liquid medium to each cartridge through the PTFE tubing system. The adsorbed analytes were eluted using two elution techniques: MeOH:CH_2_Cl_2_ (4:1) 5% FA (3 fractions of 5 mL) using the cartridge in the usual forward mode or 10%, 50% and 100% MeOH (3 fractions of 5 mL) using the cartridge in backflush mode (Figure S6). The recovered volumes were dried and concentrated in 200 µL of 50% MeOH. We calculated the 100% recovery of every CYN alkaloid by quantitating the analytes on a lyophilized sample of the filtrated culture medium. LC-DAD-MS^2^ was used to carry out the analysis in order to improve the confirmation of CYN variants at low recoveries.

Large-scale experiments using the liquid medium were performed to test the affinity of the analytes (mostly CYN and 7D-CYN) to the GNPC column when using larger sample volumes (1.5–4 L). GNPC cartridges were pretreated using 10 mL of MeOH, followed by 10 mL of water. After this conditioning step, we applied the desired volume of the liquid medium to the cartridge through the PTFE tubing system. Arbitrary volumes of 1.5, 3 and 4 L were applied once to each GNPC column. We eluted the adsorbed analytes once with 10 mL of MeOH:CH_2_Cl_2_ (4:1) 5% FA. The recovered eluates and effluents (Figure S6) were dried and concentrated in 200-µL 50% MeOH before LC-DAD-MS^2^ analysis.

### 4.3. Tests on Sample Size and Cartridge Handling for Solid Samples

Biomass experiments were intended to optimize the 7D-desulfo-CYN isolation from a complex sample. The biomass samples used in the present work consisted of lyophilized filamentous cells or cellular debris. We obtained these cyanobacterial remnants from the frozen precipitates derived from the centrifugation of dense culture broths of the *Cylindrospermopsis raciborskii* strain 11K, as stated earlier. After weighing the lyophilized sample, cells were suspended in 50% MeOH. In this suspension, cell membranes were disrupted using an ultrasonic probe sonicator. The lysate was centrifuged (10,000 rpm, 10 min, 4 °C), and the supernatant was transferred to a clean tube. The pellet was resuspended with 50% MeOH, sonicated, centrifuged and the supernatant was collected and mixed with the previous extract. The final extract was filtered through a 0.44 µm membrane and diluted with water to approximately 1% MeOH. These aqueous extracts were later permeated through the desired SPE columns through the PTFE tubing system.

The biomass experiments tested the adsorption of the analytes to the sorbents of Oasis HLB 0.5 g and C18 Sep-Pak 0.5 g cartridges (Waters^®^, Milford, MA, USA). The affinity of the analytes to the SPE sorbents while adjusting the sample’s pH (pH 4, 7 and 10) was also studied. The SPE columns were pretreated with 6-mL 100% MeOH, followed by 6 mL of water. While permeating the aqueous samples through the columns, the resulting effluent was collected and later lyophilized for analysis. We eluted the analytes from the sorbents with 6-mL 10% MeOH, followed by 6 mL pure MeOH. Both fractions were also collected for every sample. A similar experiment was performed with C18 cartridges only, in which the cartridges were eluted using 10-mL 15% MeOH, followed by 10 mL of pure MeOH (all fractions were collected for every sample).

### 4.4. Semipreparative Purification

The isolation of high-purity CYNs from the biomass or broth using SPE columns usually required this last purification step. We resuspended the GNPC extracts in Milli-Q^®^ (MilliporeSigma, Burlington, MA, USA) grade H_2_O and C18 extracts in 20% MeOH (to improve 7D-desulfo-CYN’s solubility) before the chromatographic separation using a Luna 5 µm C18 100A, 250 × 10 mm (Phenomenex^®^, Torrance, CA, USA) HPLC column. The mobile phase consisted of Milli-Q^®^ grade H_2_O, and elution of the CYN variants was achieved with a gradual increase of MeOH from 5% to 80% for 35 min. The semipreparative purification was carried out using an HPLC (LC20AD, Shimadzu Corporation, Kyoto, Japan) with a diode-array detector (SPD-M20A) coupled to a fraction collector (FRC-10A, Shimadzu Corporation, Kyoto, Japan). Detection of the CYN structural analogs was set between 200 and 600 nm using the diode-array detector.

### 4.5. Sample Preparation for LC-DAD Method Validation

A cyanobacterial strain confirmed by our lab as a nonproducer of CYN (*C. raciborskii* ITEP 18) was grown according to Section 4.1 and used as a matrix for CYN spiked-in extractions. Cultures with a density of 1 × 10^8^ cells/mL were centrifuged, and the supernatant was collected. The resulting liquid medium was divided into aliquots, each with a volume of 300 mL. Acyclovir-certified standard (Thermo Fisher Scientific, Waltham, MA, USA) was used as the IS. Calibrators and control samples were prepared using different lots of CYN-certified standards (Eurofins Abraxis, Warminster, PA, USA).

### 4.6. LC-DAD Method Validation

Validation of the LC-DAD method was performed following regulatory guidelines established by the National Agency for Sanitary Vigilance, document RDC No. 166 (Brazil 2017) [40,41]. The assessed validation parameters were linearity, limits of detection (LOD), limits of quantification (LOQ), selectivity, carryover, within-day bias and within-day imprecision.

Linearity (R^2^ ≥ 0.99) was estimated by the analysis of calibration curves (n = 5). Calibrators were prepared at the following concentrations: 0.5, 5, 25, 50, 100 and 150 µg/mL. These calibrators were considered acceptable at ≤15% coefficient of variation (CV) (≤20% CV at the lower LOQ).

Estimation of the LOD and LOQ was performed using decreasing concentrations of CYN-spiked samples. The lower LOD was the lowest concentration, with a signal/noise ratio ≥3, and the lower LOQ was the lowest concentration, with a signal/noise ratio ≥10.

Within-day imprecision and within-day bias were calculated at five concentrations in five replicates. Imprecision was expressed as the coefficient of variation (% CV). Acceptable imprecision was ≤15% CV (≤20% CV at the lower LOQ was accepted). Bias was estimated for each concentration as the relative standard error (% RSE). Relative standard errors of 85–115% were considered acceptable, and 80–120% RSE were accepted at the lower LOQ.

The matrix effect was evaluated using blank samples. Interference in the DAD detector’s response was tested at the retention times of CYN and IS (acyclovir). Interference peaks of ≤20% of the analyte response at the lower LOQ were considered acceptable. Interference peaks of ≤5% of the IS response were considered acceptable.

Chromatographic selectivity was evaluated by calculating the peak resolution (Rs) of a nonextracted mix containing the following analytes: CYN (certified standard), acyclovir (certified standard), 7D-CYN (purified fraction), 7D-desulfo-CYN (purified fraction) and desulfo-CYN. The desulfo-CYN degradation product was synthesized by diluting a purified fraction of cylindrospermopsin in an aqueous solution of HCl 5% and maintaining it in constant shaking at 70 °C during a 48-h interval.

### 4.7. LC-DAD-MS^2^ Instrumentation

The extracts were separated through a Synergi 4 µm Hydro-RP 80A, 150 × 4.6 mm (Phenomenex^®^, Torrance, CA, USA) HPLC column, protected with a guard column of the same material. The mobile phase consisted of 5-mmol/L ammonium acetate buffer with 0.05% FA. Several gradient elutions using MeOH were tested, according to Eaglesham et al. (1999) [42] and Gallo et al. (2009) [1]. The gradient elution of the final method started at a 100% aqueous mobile phase, with a gradual increase up to 80% MeOH at 15 min (flow 0.2 mL/min). Samples were resuspended in 50% MeOH, and 5 uL was injected into an HPLC (LC20AD, Shimadzu Corporation, Kyoto, Japan) with a diode-array detector (SPD-M20A, Shimadzu Corporation, Kyoto, Japan), coupled to a quadrupole time-of-flight mass spectrometer (Micro TOF-QII; Bruker Daltonics, Billerica, MA, USA) with an ESI (electrospray ionization) interphase.

The detection range for CYNs’ quantitation was set between 200 and 600 nm (λ_max_ = 262 nm) using the diode-array detector. Since the diode-array detector was coupled to the Micro TOF-QII analyzer, quantitation and structure confirmation of CYN and structural analogs were carried out simultaneously. The ionization source conditions were as follows: capillary potential of 3500 V, temperature and flow of drying gas (nitrogen) of 300 °C and 5 mL/min, respectively, and a nebulizer pressure of 35 psi. Mass spectra were acquired using electrospray ionization in the positive mode, *m*/*z* range 50 to 3000. The Micro TOF-QII instrument was operated in MS^1^ scan mode or MS^2^ MRM (multiple reaction monitoring) mode as needed. In the MRM mode, CID (collision-induced dissociation) experiments were performed using the following collision energies for fragmentation: 20 eV, 35 eV and 40 eV.

### 4.8. Toxicity of the CYN Variants

HepG2 cells (Code 0103) purchased from Rio de Janeiro Cell Bank (BCRJ, Rio de Janeiro, Brazil) were grown in DMEM (Dulbecco’s Modified Eagle’s medium) high glucose (Gibco^®^, Thermo Fisher Scientific, Waltham, MA, USA) supplemented with 10% fetal bovine serum (FBS), 100 U/mL penicillin and 0.1 µg/mL streptomycin at 37 °C in a 5% CO_2_ atmosphere. HepG2 cells were seeded in 6-well plates with a density of 2.5 × 10^5^ cells/well, incubated overnight and treated with the chosen CYN variant at the desired time interval. After the treatment intervals, cells were rinsed with phosphate-buffered saline (PBS). The cells were detached from the flask using 300 µL of 2.5 g/L trypsin solution (0.25% trypsin diluted in PBS) for 5 min. DMEM with 10% FBS (3 mL) was added to inactivate the trypsin, transferred to the collection tubes and centrifuged at 250× *g* for 5 min at 4 °C. The supernatant was discarded, and the pellet was washed using PBS with 4% FBS, followed by 200 µL of annexin V-binding buffer (10 mM Hepes pH 7.4, 140 mM NaCl and 2.5 mM CaCl_2_). We stained the cells using 5 µL of annexin V conjugated with allophycocyanin (annexin V-APC) (BD^TM^, East Rutherford, NJ, USA) and 2 µL of propidium iodide (PI) (Sigma-Aldrich, St. Louis, MO, USA). The samples were incubated for 15 min in the dark at room temperature. Cells treated with 10% dimethyl sulfoxide and with 40% methanol were used as positive controls for apoptosis and necrosis, respectively, and cells without any treatment were used as the negative control. We analyzed the samples on a flow cytometer FACS Canto II (BD^TM^, East Rutherford, NJ, USA), acquiring 10,000 events using the FACS Diva™ software, version 8.0 (BD^TM^, East Rutherford, NJ, USA). The excitation wavelength was λ_EX_ 633 nm and 480 nm and emission at λ_EM_ 660 nm and 578 nm for annexin V-APC and PI, respectively. The samples were analyzed using FlowJo software version 8.0 (BD^TM^, Ashland, OR, USA). The analysis was conducted in quadruplicate, and the results were expressed as mean ± the standard deviation. Statistical comparisons between groups (control vs. treatment) were performed using Mann-Whitney U tests; *p* < 0.05 was considered statistically significant.

## Figures and Tables

**Table 1:** Mean measured and calculated monoisotopic *m*/*z* value (including the mean error) of the protonated precursor ion (M + H^+^) of each of the isolated cylindrospermopsins. All measures were performed on a QTOF mass analyzer.

Analyte	Mean Measured M + H^+^ (*m*/*z*) n = 3	Calculated M + H^+^ (*m*/*z*)	Mean Error (ppm)
CYN	416.1235	416.1240	1.2
7D-CYN	400.1288	400.1291	0.87
7D-desulfo-CYN	320.1728	320.1723	1.6

## Supplementary Materials

The following are available online. Table S1: Calculated log P of CYN structural analogs according to three different cheminformatics tools. Figure S1: Sample’s effluent from C18 (red chromatogram) or from GNPC (green chromatogram) cartridges. Figure S2: Sample elution using 5-mL 10% MeOH from C18 (red chromatogram) or from GNPC (green chromatogram) cartridges. Figure S3: Sample elution using 5-mL 50% MeOH from C18 (red chromatogram) or from GNPC (green chromatogram) cartridges. Figure S4: Sample elution using 5-mL 75% MeOH from C18 (red chromatogram) or from GNPC (green chromatogram) cartridges. Figure S5: Sample elution using 5-mL MeOH from C18 (red chromatogram) or from GNPC (green chromatogram) cartridges. Table S2: Percent recovery comparison of CYN, 7D-CYN and 7D-desulfo-CYN on GNPC cartridges using two elution techniques: MeOH:CH_2_CL_2_ (4:1) 5% FA using the cartridge on the usual forward mode (3 fractions of 5 mL) or 10%, 50% and 100% MeOH on cartridge-backflush mode (3 fractions of 5 mL). Figure S6: Sample’s effluent from a GNPC cartridge acquired on a LC-DAD-MS^2^. Figure S7: Assay linearity in liquid medium samples analyzed on a LC-DAD. Table S3: Selected analytical performance parameters, criteria and experimental results for the final method. Table S4: Criteria, experimental data and status for within-day precision and bias of 5 levels of quality control liquid medium samples (n = 5). Figure S8: Chromatographic separation of CYN and four similar analytes on a Synergi Hydro-RP (C18) column acquired in a LC-DAD-MS^2^.
