# Supplementary material for: Alternative Isolation Protocol for Desulfo and Zwitterionic Cylindrospermopsin Alkaloids and Comparison of Their Toxicity in HepG2 Cells
Source: Molecules. 2020 Jul 2;25(13):3027. doi: 10.3390/molecules25133027 (PMC7412431; doi:10.3390/molecules25133027)
Supplement: Supplementary file 1 [file molecules-25-03027-s001.pdf]

molecules

MDPI

# Supplementary Materials

# Alternative isolation protocol for desulfo and zwitterionic cylindrospermopsin alkaloids and comparison of their toxicity in HepG2 cells

**Carlos González, Felipe Dörr, Renata Albuquerque, Janice Onuki and Ernani Pinto**

**Table S1.** Calculated log P of CYN structural analogs according to three different cheminformatics tools.

| CYN Variant | Calculated logP | | | | |
|---|---|---|---|---|---|
| | Chemicalize[1] | Molinspiration[2] | VCCLAB[3] | SwissADME[4] | Average |
| CYN | -2.65 | -3.66 | -1.72 | -1.77 | -2.45 |
| Epi-CYN | -2.65 | -3.66 | -1.72 | -1.77 | -2.45 |
| desulfo-CYN | -3.57 | -1.34 | -1.11 | -0.88 | -1.73 |
| 7D-CYN | -1.96 | -2.67 | -1.37 | -0.95 | -1.74 |
| 7D-desulfo-CYN | -2.90 | -0.35 | -0.44 | -0.1 | -0.95 |
| 7D-desulfo-acetyl-CYN | -2.46 | 0.36 | 0.06 | 0.32 | -0.43 |
| chloro-CYN | -1.99 | -3.06 | -1.32 | -1.20 | -1.89 |
| cylindrospermic acid | -1.85 | -3.50 | -1.68 | -1.79 | -2.21 |

[1] Chemicalize © ChemAxon Ltd.
[2] Molinspiration © Molinspiration Cheminformatics
[3] ALOGPS 2.1 © Virtual Computational Chemistry Laboratory (VCCLAB)
[4] SwissADME © Swiss Institute of Bioinformatics ©

Figures S1 to S5 were acquired in a LC-DAD using method described in Materials and Methods 4.4. Figure S1 shows breakthrough of CYN and 7D-CYN in the sample's effluent recovered from the C18 SPE column (red chromatogram). This indicates a limited capacity of the C18 cartridges to adsorb CYN and 7D-CYN at the tested conditions. In contrast, no CYN analogs were detected on the effluent recovered from the GNPC columns (green chromatogram), as shown in Figure S1. According to these qualitative results, the GNPC cartridge had higher affinity for CYN and 7D-CYN.

molecules

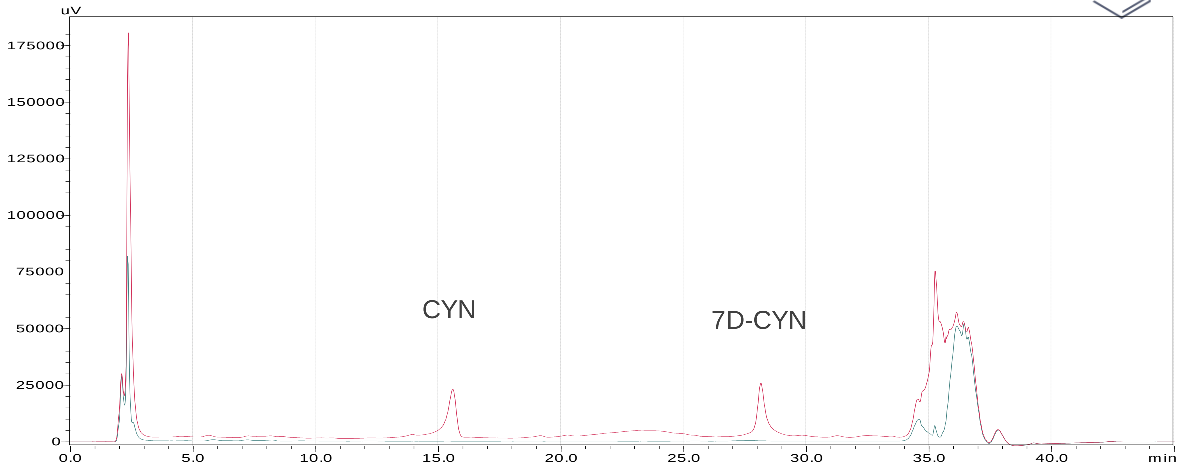


Retention times for the main analytes: CYN 15 min, 7D-CYN 28 min.
*The samples consisted of 300 mL of a dense *C. raciborskii 11K* strain culture. The culture broth was centrifuged, filtered and finally applied through the respective SPE cartridge. The total effluent was collected, and 50 mL were lyophilized, concentrated and analyzed (n= 2).

**Figure S1.** Sample's* effluent from C18 (red chromatogram) or from GNPC (green chromatogram) cartridges. The overlapped chromatograms acquired on a LC-DAD, show the extracted signal at $\lambda$ 262 nm.

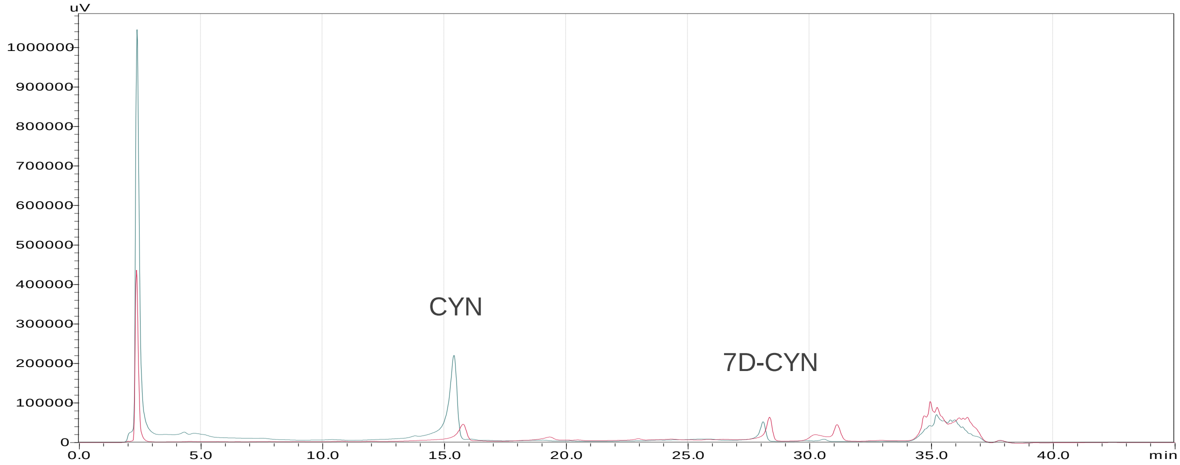


Retention times for the main analytes: CYN 15 min, 7D-CYN 28 min.
*The samples consisted of 300 mL of a dense *C. raciborskii 11K* strain culture. The culture broth was centrifuged, filtered and finally applied through the respective SPE cartridge. The eluate was collected, concentrated and analyzed (n= 2).

**Figure S2.** Sample* elution using 5 mL 10% MeOH from C18 (red chromatogram) or from GNPC (green chromatogram) cartridges. The overlapped chromatograms acquired on a LC-DAD, show the extracted signal at $\lambda$ 262 nm.

molecules

MDPI

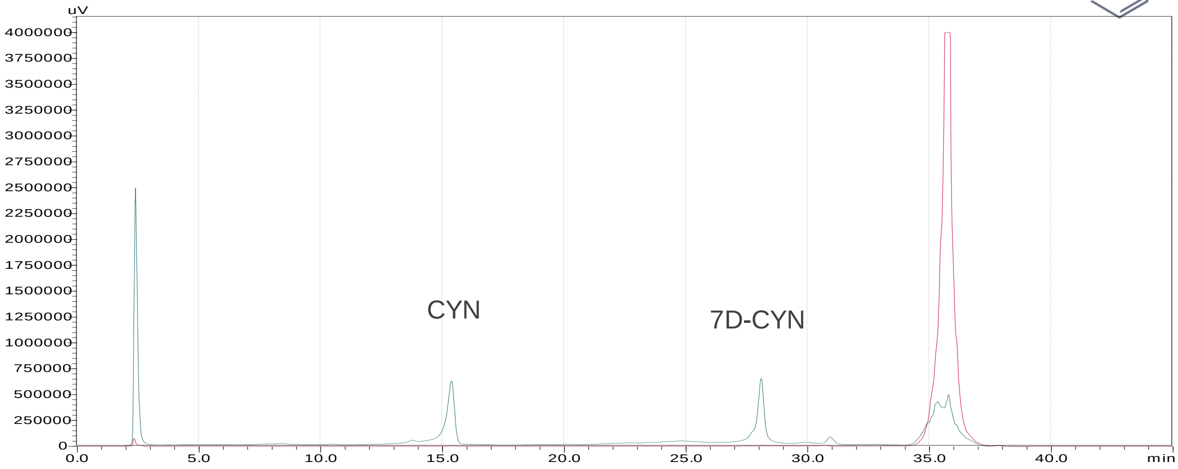


Retention times for the main analytes: CYN 15 min, 7D-CYN 28 min.
*The samples consisted of 300 mL of a dense *C. raciborskii 11K* strain culture. The culture broth was centrifuged, filtered and finally applied through the respective SPE cartridge. The eluate was collected, concentrated and analyzed (n= 2).

**Figure S3.** Sample* elution using 5 mL 50% MeOH from C18 (red chromatogram) or from GNPC (green chromatogram) cartridges. The overlapped chromatograms acquired on a LC-DAD, show the extracted signal at $\lambda$ 262 nm.

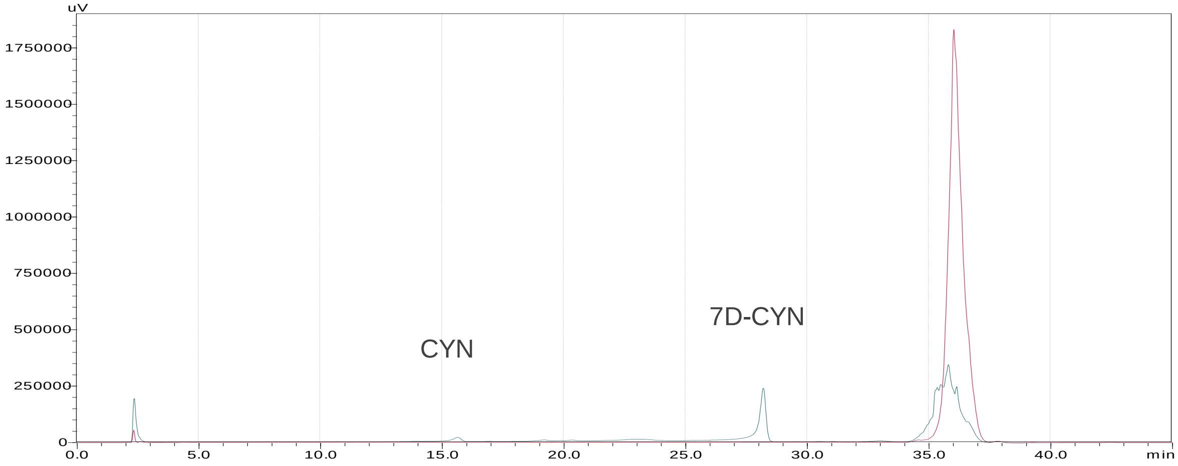


Retention times for the main analytes: CYN 15 min, 7D-CYN 28 min.
*The samples consisted of 300 mL of a dense *C. raciborskii 11K* strain culture. The culture broth was centrifuged, filtered and finally applied through the respective SPE cartridge. The eluate was collected, concentrated and analyzed (n= 2).

**Figure S4.** Sample* elution using 5 mL 75% MeOH from C18 (red chromatogram) or from GNPC (green chromatogram) cartridge. The overlapped chromatograms acquired on a LC-DAD, show the extracted signal at $\lambda$ 262 nm.

molecules

MDPI

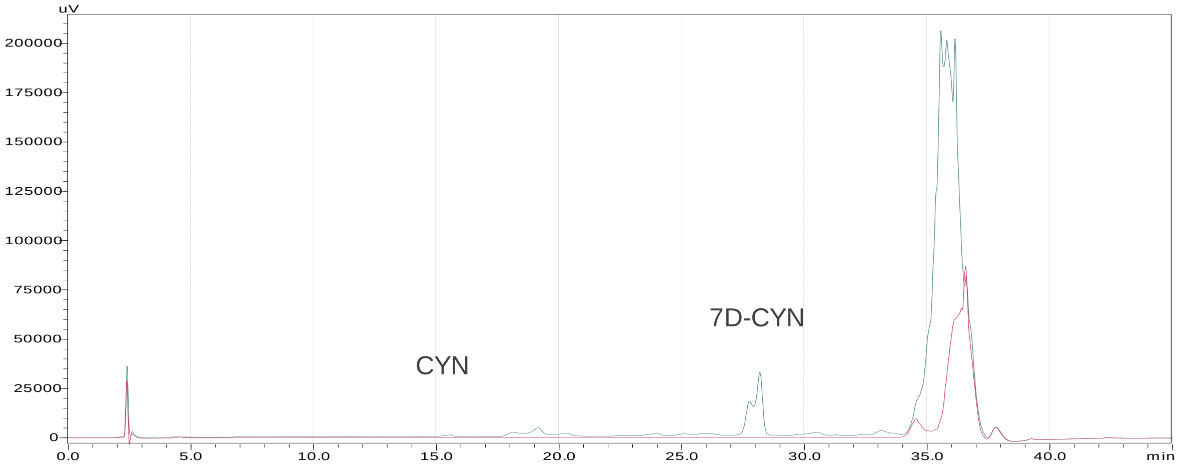


Retention times for the main analytes: CYN 15 min, 7D-CYN 28 min.
*The samples consisted of 300 mL of a dense *C. raciborskii 11K* strain culture. The culture broth was centrifuged, filtered and finally applied through the respective SPE cartridge. The eluate was collected, concentrated and analyzed (n= 2).

**Figure S5.** Sample* elution using 5 mL MeOH from C18 (red chromatogram) or from GNPC (green chromatogram) cartridges. The overlapped chromatograms acquired on a LC-DAD, show the extracted signal at $\lambda$ 262 nm.

**Table S2.** Percent recovery comparison of CYN, 7D-CYN and 7D-desulfo-CYN on GNPC cartridges using two elution techniques: MeOH:CH2CL2 (4:1) 5% FA using the cartridge on the usual forward mode (3 fractions of 5 mL); or 10%, 50%, and 100% MeOH on cartridge backflush mode (3 fractions of 5 mL). Data was acquired on a LC-DAD-MS² instrument, and quantitation of the CYNs was performed at $\lambda$ 262 nm (n= 2).

| Elution method used on the graphitized carbon cartridge | Recovered Fraction from cartridge | Percent recovery (%) | | | | | | | | | | | |
|---|---|---|---|---|---|---|---|---|---|---|---|---|---|
| | | CYN | | | | Deoxy-CYN | | | | Desulfo-deoxy-CYN | | | |
| | | Replicate 1 | Replicate 2 | SD | RSD | Replicate 1 | Replicate 2 | SD | RSD | Replicate 1 | Replicate 2 | SD | RSD |
| Methanol-Dichloromethane 4:1, 5% Formic Acid | 1st Fraction | 100.52 | 98.02 | 1.77 | 1.18 | 34.39 | 28.32 | 4.29 | 8.83 | 72.75 | 75.45 | 1.91 | 1.73 |
| | 2nd Fraction | 1.12 | 0.79 | 0.23 | 15.35 | 40.93 | 54.25 | 9.42 | 13.84 | 0.00 | 2.54 | 1.80 | 141.42 |
| | 3rd Fraction | 0.00 | 0.00 | 0.00 | NaN | 3.91 | 0.00 | 2.76 | 70.71 | 0.00 | 0.00 | 0.00 | NaN |
| | Total | 101.64 | 98.81 | 2.00 | 1.32 | 79.22 | 82.57 | 2.37 | 1.97 | 72.75 | 77.99 | 3.71 | 3.32 |
| 10%, 50%, 100% MeOH-Water (Cartridge on Backflush) | 1st Fraction (10% MeOH) | 40.65 | 36.12 | 3.20 | 5.46 | 22.44 | 24.13 | 1.19 | 3.46 | 12.00 | 9.01 | 2.11 | 12.81 |
| | 2nd Fraction (50% MeOH) | 46.61 | 49.51 | 2.05 | 2.88 | 52.90 | 61.70 | 6.23 | 7.43 | 81.78 | 79.84 | 1.37 | 1.13 |
| | 3rd Fraction (100% MeOH) | 0.89 | 1.03 | 0.10 | 6.78 | 8.39 | 0.74 | 5.41 | 61.75 | 0.00 | 2.45 | 1.73 | 141.42 |
| | Total | 88.15 | 86.66 | 1.06 | 0.80 | 83.73 | 86.57 | 2.01 | 1.58 | 93.78 | 91.30 | 1.75 | 1.26 |

NaN: not a number, SD: standard deviation, RSD: relative standard deviation.

molecules MDPI

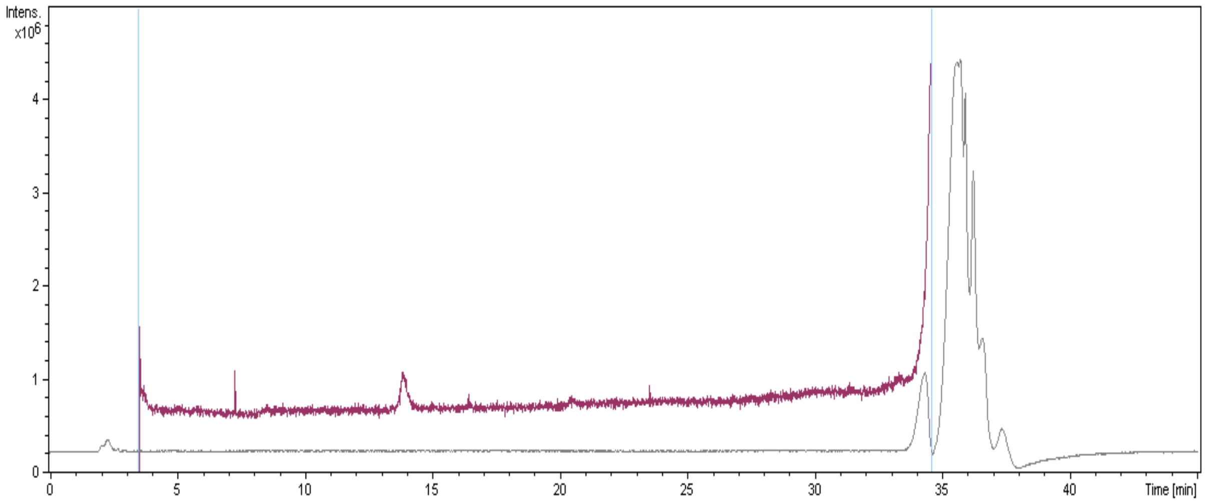


* The sample consisted of 4 L of a dense C. raciborskii 11K strain culture. The culture broth was centrifuged, filtered and finally applied through the respective SPE cartridge. The total effluent was collected, and 500 mL were lyophilized, concentrated and analyzed (n= 2).

**Figure S6.** Sample's* effluent from a GNPC cartridge acquired on a LC-DAD-$MS^2$. The overlapped chromatograms show the extracted signal at $\lambda$= 262 nm (grey chromatogram) and TIC for MS1 (purple chromatogram). No $M+H^+$ precursor ions corresponding to the m/z of the CYN variants were detected on this run (purple chromatogram).

Large scale experiments using liquid medium were performed to test the affinity of the analytes (mostly CYN and 7D-CYN) to the GNPC column when using volumes between 1.5 – 4 L. Figure S6 displays results from a 4 L sample's effluent using a GNPC cartridge (data acquired on a LC-DAD-$MS^2$). No protonated precursor ions corresponding to the m/z of the CYN variants were detected. These results indicate complete adsorption of the analytes to the carbon SPE column even after applying four liters of filtered culture broth. A successful elution of all the analytes from the carbon cartridge was achieved by eluting once with 10 mL of MeOH:$CH_2Cl_2$ 4:1 with 5% FA.

The definitive total recovery protocol proposed by our group is shown in Figure 2.

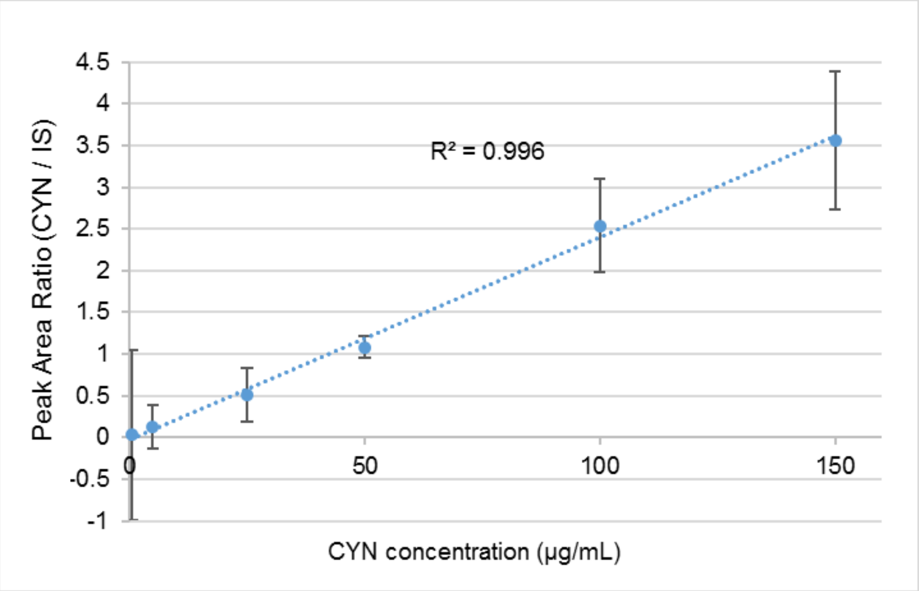


RSD= relative standard deviation

**Figure S7.** Assay linearity in liquid medium samples analyzed on a LC-DAD. As an extraction protocol we used Figure 18 steps 1b to 7b. Variation bars indicate calibrator's % CV , multiplied by 10 for visualization (n= 5).

**Table S3.** Selected analytical performance parameters, criteria, and experimental results for the final method. Limits of detection and quantitation were calculated by spiked sample dilution.

| Parameters | Criteria | Experimental data |
|---|---|---|
| **Linearity** | R ≥ 0.990 | R = 0.996 |
| **Calibration curve data deviation** | RSD % ≤ 15 * | RSD % = 1.3 – 10.2 |
| **Lower limit of quantitation** | 1 µg/L [α] | 0.2 µg/L |
| **Limit of detection** | N.A. | 0.04 µg/L |

RSD %= percent relative standard deviation, R= correlation coefficient
* Ministry of Health, National Agency for Sanitary Vigilance, Regulatory Guidelines RDC No. 166, Brazil 2017.
[α] Ministry of Health, Consolidated Regulatory Guidelines No. 5, Annex XX, Brazil 2017.

molecules

MDPI

**Table S4.** Criteria, experimental data, and status for within-day precision and bias of 5 levels of quality control liquid medium samples (n= 5).

| Quality Control Sample (µg/mL) | Within-day Precision (% CV) | | | Within-day Bias (% RSE) | | |
|---|---|---|---|---|---|---|
| | Criteria* | Experimental | Status | Criteria* | Experimental | Status |
| 0.5 | ≤ 20 | 7.5 | Accepted | 80 – 120 | 115 | Accepted |
| 1.5 | ≤ 15 | 1.9 | Accepted | 85 – 115 | 98 | Accepted |
| 75 | ≤ 15 | 1.6 | Accepted | 85 – 115 | 100 | Accepted |
| 120 | ≤ 15 | 1.2 | Accepted | 85 – 115 | 95 | Accepted |
| 250 | ≤ 15 | 6.2 | Accepted | 85 – 115 | 90 | Accepted |

%CV= coefficient of variation, %RSE= relative standard error
*Ministry of Health, National Agency for Sanitary Vigilance, Regulatory Guidelines RDC No. 166, Brazil 2017.

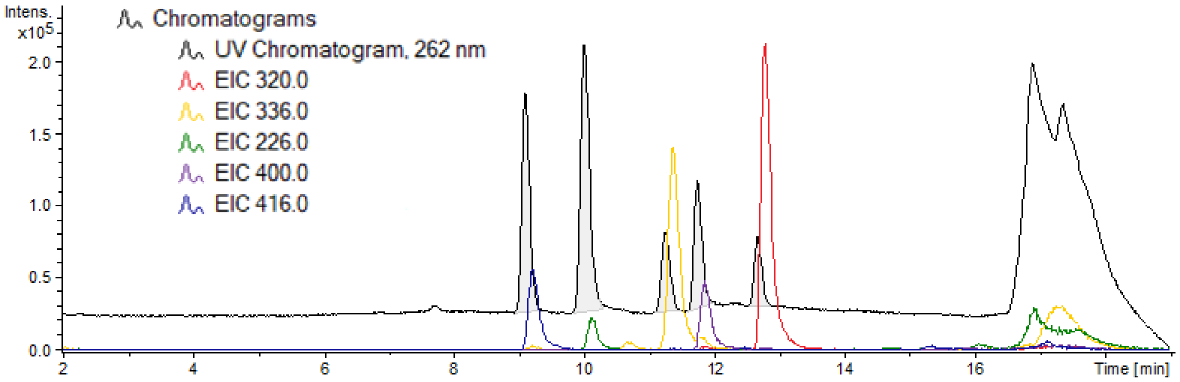


**Figure S8.** Chromatographic separation of CYN and four similar analytes on a Synergi Hydro-RP (C18) column, acquired on a LC-DAD-MS$^2$. The black chromatogram shows the extracted signal at a wavelength ($\lambda$) of 262 nm, and the colored chromatograms show the extracted ion chromatogram (EIC) for the protonated precursor's m/z of each analyte. The retention times (min) for the five compounds are CYN, 9.1 (blue); acyclovir, 10.0 (green); desulfo-CYN, 11.3 (yellow); 7D-CYN, 11.7 (purple); and 7D-desulfo-CYN, 12.7 (red).
